# EUFEMED London Conference 2017: Exploratory Medicines Development: Innovation and Risk Management

**DOI:** 10.3389/fphar.2017.00901

**Published:** 2018-01-17

**Authors:** Luc Van Bortel, Hildegard Sourgens, Kerstin Breithaupt-Grögler, Henri Caplain, Yves Donazzolo, Ingrid Klingmann, Michael Hammond, Timothy C. Hardman, Steffan Stringer, Jan de Hoon

**Affiliations:** ^1^Belgian Association of Phase-I Units, Antwerp, Belgium; ^2^Association for Applied Human Pharmacology, Hamburg, Germany; ^3^Club Phase-I, Paris, France; ^4^Association for Human Pharmacology in the Pharmaceutical Industry, London, United Kingdom

**Keywords:** early phase clinical drug development, innovation in drug development, risk mitigation, conference report, the EUropean Federation for Exploratory MEdicines Development (EUFEMED)

## Abstract

The first formal conference of the EUropean Federation for Exploratory MEdicines Development (EUFEMED) held in London was the result of a collaborative effort of its founding associations: the Association for Applied Human Pharmacology (AGAH; Germany), the Association for Human Pharmacology in the Pharmaceutical Industry (AHPPI; UK), the Belgian Association of Phase-I Units (BAPU; Belgium), and Club Phase-I (France). The conference focused on innovation and risk management in early clinical drug development. Among other innovations, immunotherapy in oncology and inflammatory diseases were discussed as well as the importance of adaptive trial designs in early clinical drug development. Consideration was given to assessing and mitigating risk in early clinical drug development, and included a preconference workshop. Different measures to minimize risks in healthy volunteers and patients in first-in-human trials were discussed in addition to the importance of non-clinical data, the need for reliable biomarkers, improved communication on adverse events (AEs) and well-trained study sites with ready access to intensive care units and clinical specialists. The need for a European-wide system for prevention of over-volunteering was also discussed. The conference provided opportunity to discuss these developments and concerns and the changing regulatory environment with stakeholders from academia, industry, and regulatory agencies including the European Medicines Agency (EMA). Presentations given by invited speakers are published on http://www.eufemed.eu/london-conference-2017/.

## Introduction

The EUropean Federation for Exploratory MEdicines Development (EUFEMED, www.eufemed.eu) is a not-for-profit association that aims to improve the early phase clinical drug development process in Europe. Its first formal conference held in London focused on innovation and risk management. This report summarizes the key learnings from an audience perspective derived from the conference.

## Preconference 1-day workshop

### Practical aspects of assessing and mitigating risk in early phase clinical trials

The EUFEMED president, Jan de Hoon (University Hospital Leuven, Belgium) welcomed attendees and explained how the focus of the workshop topic was a consequence of the Bial incident in France (Enserink, 2016).

Stephanie Plassmann (Preclinical Safety Consultants, Switzerland) emphasized the key role of the toxicologist during drug development, how timely communication between all disciplines is mandatory and how non-clinical and clinical development remain closely intertwined. She concluded that ongoing risk assessments should integrate all data as they become available, including those from the public domain.

Friedemann Schmidt (Sanofi, Germany) described computational systems toxicology and *in silico* prediction of off-target activities and how data derived from integrated *in silico* methods are gaining regulatory acceptance. As it is impossible to screen for all off-target effects and since all methods have their limitations, combining methods can provide a greater spread of information.

Marc Pallardy (Institut national de la santé et de la recherche médicale, INSERM, University Paris-Sud, France) spoke about determination of the first dose for multispecific monoclonal antibodies. Side effects from the broad range of biologics in development are very difficult to predict. Low doses have been implemented in some clinical trials, but these doses are far from clinically active levels.

Bruno Boutouyrie-Dumont (Novartis, Switzerland) discussed dose selection based on the minimal anticipated biological effect level (MABEL) (CHMP, 2007). It was concluded how the first-in-human dose should be based on available non-clinical data and on different estimates. Safety assessment should take into account pharmacodynamic (PD) activity and the duration of action in man. Protocol development should be a team collaboration and should, these days, consider the potential for employing an adaptive design.

Philippe Grosjean and Eric Legangneux (Sanofi, France) challenged the usefulness of the maximum tolerated dose (MTD) and its value in developing medicines for humans. It was questioned why doses must be escalated so high; why take the risk? Is this approach ethical?

Workshop attendees were privileged to hear from representatives of regulatory agencies from UK, Belgium, and Germany. Thomas Sudhop (Bundesinstitut für Arzneimittel und Medizinprodukte, BfArM, Germany) provided a regulatory update on the new EU guideline on First-in-Human clinical trials (CHMP, 2017). The 2007 guideline (CHMP, 2007) was intended for risk mitigation with high-risk investigational medicinal products (IMPs) and only covered single ascending dose administration. The updated guideline covers more complex designs and also studies in patients. With the Bial trial in mind, the guideline was developed with safety as the underlying principle. The guideline should not be seen as mandatory. But where alternative strategies are requested, full scientific justifications should be provided.

## Day 1

### Keynote: incidents happen—which lessons can we learn?

Jan de Hoon focused on how incidents have shaped drug regulation. Most recently, the death of a healthy volunteer in the Bial trial (Enserink, 2016) led to discussions on how future trials should be conducted. Over the past 50 years, 12 deaths have been reported in Phase-I trials worldwide; five were possibly drug-related, equating to a risk of death of 1 in 500,000 subjects. To minimize risks, investigators and those working in industry should have appropriate competence and experience. There should be improved communication, independent expert review of data, and measures to prevent over-volunteering. It may also be advisable to increase the number of inspections conducted by agencies and the level of accreditations required by sites and investigators.

### Session 1: managing risks in early phase clinical trials

Ulrike Lorch (Richmond Pharmacology, United Kingdom, UK) spoke about the revision of the EMA's guideline on strategies to identify and mitigate risks for early clinical trials (CHMP, 2017). When applying the guideline, one should be proportionate to uncertainty and potential risks, avoid getting stuck in marginal issues, allow for further investigations where appropriate and use simple algorithms for potentially fundamental risks. Knowledge, expertise and an expert team are essential. Training and clinical pharmacology unit accreditation schemes should be considered, as well as obtaining scientific advice pre-Clinical-Trial-Authorization (CTA) submission.

Annick Peremans (Research Centre Aalst, Belgium) asked how a European-wide system could be established to prevent over-volunteering. Some healthy volunteers travel from one country to another, risking drug interactions, uncontrolled radiation exposure, and unknown consequences for their future health. There are currently two national databases in Europe: UK (TOPS) and France (VRB). Belgium/Germany/Netherlands use a privately owned database (VIPCHECK), but this database is not used by all institutions. Only a European subject database would be able to raise the ethical standards and safety of clinical research in Europe.

### Session 2: scientific tools in early development of medicines to mitigate risk

Philippe Danjou (Biotrial, France) discussed minimizing risk in early development of central-nervous-system (CNS) medicines in the context of the death of a healthy volunteer in the Bial trial, where the subject was over 50 years of age and had an underlying pathology that was only detectable on autopsy. The first option would be to monitor the primary effect or target occupancy through molecular imaging. However, this is impossible when relying on animal data alone. Additional barriers include the availability of human ligands and cost. If molecular imaging is not possible, a downstream biomarker is another option, although the current state of knowledge and validation of assays may prove to be difficult. Conducting trials solely in a patient population is a third but unrealistic option. Healthy subjects frequently tolerate and overcome AEs and toxicities better than older, co-medicated patients with multiple pathologies.

An Van den Bergh (Janssen, Belgium) noted that the utility of physiology-based pharmacokinetics in risk mitigation in early studies lies in predicting the time profile of drug concentrations based upon *in vitro* absorption, distribution, metabolism, and excretion data along with a plausible biology. It can also confirm mechanisms governing (non-linear) pharmacokinetics during dose escalation in the first-in-human ascending dose studies. It provides an ability to anticipate the impact of genetic polymorphisms on the pharmacokinetic profile and to anticipate drug interactions.

Elaine Holmes (Imperial College London, UK) spoke about the interaction of an individual's genetic blueprint, the genetics of the individual's microflora, and the environment, which could take the form of food, food additives, drugs, and contaminants. It is becoming clear that the microbiome plays an important role in human health. Metabonomic profiling of biofluids (urine, stool, plasma, vaginal and oral swabs) will help elucidate the functionality of the microbiome and identify associations with disease and disease risk.

### Session 3: posters and oral presentations

Researchers were given the opportunity to submit abstracts to be displayed as posters. A selection was presented as oral presentations. All abstracts are published in http://www.frontiersin.org/books/EUFEMED_2017/1195.

### Session 4: examples of innovation and risk management

Jorg Taubel (Richmond Pharmacology, UK) described how integrated adaptive Phase-I clinical trials are safer, take less time and cost less than traditional alternatives. He advised that adaptive designs can establish a “playing field” with set boundaries on various factors including starting dose, maximum exposure limits, numbers of subjects, procedures, samples, and ‘inconveniences.’ They also provide flexibility within the ‘playing field's’ boundaries to respond to challenges arising during the trial, all overseen by a safety review committee. Considering the worst case scenario, adaptive designs are possible with careful planning and expertise on the part of all stakeholders.

David Jones (Medicines & Healthcare products Regulatory Agency, MHRA, UK) explained that it was considered necessary to revise the 2007 Risk Mitigation guidelines (CHMP, 2007) after review of the Bial incident. The revision addresses the evolution of practices in first-in-human clinical trials including the increasing trend to use integrated protocols. The guideline emphasizes the critical value of pharmacology and understanding the mode of action of an IMP. Emphasis should be placed on estimation of the exposure at the initial dose and following dose escalations to a predefined maximum exposure. The starting dose for healthy volunteers should be below the expected pharmacologically active dose, unless a robust justification for a higher dose can be provided. The protocol should define the maximal level of exposure, which is usually based on the ‘no observed adverse effect level’ (NOAEL). By moving from single to multiple dosing, selection of dosing intervals and durations should take into account the specific pharmacokinetic and pharmacodynamic characteristics of the IMP.

Christopher Goldring (University of Liverpool, UK) presented innovative *in vitro* models of toxicology assessments of drug-induced liver injury. Increasingly complex model systems were presented ranging from single cell two-dimensional systems, such as primary hepatocytes and liver-derived cell lines, to three-dimensional systems, such as multi-cell culture and liver spheroids. A battery test system can be developed with appropriate refinement and benchmarking. Multi-dimensional *in vitro* systems with a relevant physiological and pharmacological phenotype are also being progressed.

Alan Boyd (Royal College of Physicians, UK) provided some examples of innovation and risk management. These included a gene therapy based product (Cerepro® in malignant glioma), molecule repurposing (ACE inhibitors in cancer cachexia), and an immunotherapy (a bifunctional biologic for treatment of malignant melanoma). Interestingly, none of these examples gained marketing authorization. Alan Boyd reported how the number of new drug applications has been declining steadily over the last 15 years and concluded that it is crucial to advance new strategies to reduce the development time frame, decrease costs, and improve success rates.

## Day 2

### Session 5: assessment and mitigation of risk in modern development strategies for paediatrics

Saskia de Wildt (Radboud University Nijmegen, The Netherlands) gave some historical examples of how poorly we understand the absorption, metabolism, and clearance of medicines in children. The benefits of ^14^C microdosing were discussed, covering misplaced concerns over exposure to radioactivity. It was concluded that although there are challenges yet to be addressed with microdosing, it has the potential to provide critical information that will empower a more informed approach to medicine development in children.

An Oxford debate addressed the question of whether the development of pediatric medicines should be limited to pharmacokinetic bridging trials alone. The motion was defended by Clair Aubrey (GlaxoSmithKline, GSK, UK) and Christoph Male (Medical University Vienna, Austria) countered the motion. The audience felt that pharmacokinetic bridging can be useful to predict the dose, but this does not necessarily extrapolate to the effect a drug may have. Overall, using all the information available was felt the optimal way to determine the best dose to be used in children.

### Parallel workshops

#### Workshop 1: how to use the results from non-clinical studies to better predict the risks in early phase clinical trials? moderator: stephanie plassmann

An integrative approach is needed when considering the various data from non-clinical testing. Consideration was given to the values and limitations of commonly determined parameters such as the MTD, NOAEL, and safety ratio as well as pharmacokinetic parameters. Emphasis was also placed on contextualizing findings. The example was that of patients with neurologic diseases who often tolerate considerably higher doses than healthy subjects. Finally, an exercise on choosing the starting dose based on non-clinical data was given. Safety margins should take into account possible differences in pharmacokinetics (such as drug absorption and protein binding) and pharmacodynamics (including susceptibility to develop AEs), between the animal species and humans.

#### Workshop 2: modern drug development in oncology—how to successfully design the early phase trials? moderators: heike oberwittler (ipsen, france) and sylvie rottey (ghent university, belgium)

The changing face of oncology studies was reviewed in the context of adaptive trial designs, the use of biomarkers and novel immunotherapies. It was considered how designing trials intelligently can facilitate the smooth transition through dose selection, expansion cohorts and indications—all within a single adaptive-design Phase-I trial. Development is enhanced if biomarkers are identified to monitor the activity of a drug. It might be possible to further refine the study population with genetic testing, where markers of specific susceptibility can be determined. Identifying the right dose for cancer patients remains challenging. Should it be dependent on the size of the tumor? Labeling and imaging techniques can better identify drug distribution and delivery to the target site.

The increasing number of clinical trials and targets being developed are making recruitment of patients to test new agents increasingly challenging. The possibility of including healthy volunteers remains limited. In all cases the value of the trial's outcome is limited by how dose-limiting toxicities and anti-tumor activity are assessed.

#### Workshop 3: incident management in phase-i trials: what to do if things go wrong? moderators: katherina erb-zohar (clinphase; germany) and yves donazzolo (eurofins, france)

Focus was first placed on ICH E6(R2), 2016 and its requirements that foreseeable risks and inconveniences are weighed against. A trial should be initiated and continued only if the anticipated risks are and remain acceptable. It is essential to be prepared for a critical situation, involve staff experienced in managing emergencies and collaborate with local intensive care providers. The principal investigator or qualified delegate must always be available and well-informed of the IMP and the trial, which should incorporate appropriate stopping rules. It is also advised that units build relationships with local specialists to facilitate the provision of support “on-demand” (cardiologist, neurologists, psychiatrist, etc.). When things do go wrong, it is essential to manage incidents proactively. Take steps to avoid symptom progression and prevent harm to other subjects, follow applicable reporting procedures for serious AEs and apply appropriate stopping rules. And finally, once the clinical aspects of the incident have been managed, it is useful to involve professionals to document and share the lessons learned.

### Session 6: assessment and mitigation of risk in trials with biologicals

Christian Blank (The Netherlands Cancer Institute, The Netherlands) took the audience on a historical journey from the 1890s and the ‘fathers’ of the tumor immunology concept to the early 2000s, covering the theory of immune-surveillance, the start of clinical trials with anti-‘cytotoxic T lymphocyte-associated antigen 4’ (CTLA-4) molecules and improved survival of patients with metastatic melanoma with ipilimumab. The action of the “programmed death-ligand 1” (PD-L1) and its upregulation induced by interferon-producing tumor-infiltrating CD8 T cells was used to introduce the concept of adaptive immune resistance, the cancer immunogram, and personalized immunotherapy. The presentation concluded that combinations of checkpoint inhibitors with short-term targeted therapies will be the approach most likely to prove successful in the future.

Ioanis Karydis (University of Southampton, UK) looked at mitigating immunotoxicity in early phase trials in oncology. Animal models used to predict toxicity are problematic as they do not necessarily reflect the host system and there is a pressing need for predictive biomarkers. Patients are usually highly motivated and well-informed as well as prepared to accept significant risk of morbidity and mortality. Consequently, there is a potential to under-report adverse events. In addition, patients usually have been heavily pre-treated and express long-term toxicities, making monitoring challenging. Appropriate care should be taken not only in designing trials, preferably involving adaptive designs, but also in selecting study sites. These sites should have competence and experience in dealing with immune response AEs, and sufficient resources to deal with the increased burden associated with adaptive/multi-arm clinical trials, and with a high level of cross-speciality support.

Ann Gils (Catholic University of Leuven, Belgium) reviewed immunotoxicity of IMPs for inflammatory disease. Monitoring of IMP concentrations using enzyme-linked immunosorbent assays (ELISA), factors influencing their pharmacokinetics and the incidence of anti-drug antibodies (ADA) were reviewed. Consideration was given to the differences between non-neutralizing and neutralizing ADAs, the development of drug tolerant ADA assays and symptomatic AE profiles associated with ADA detection using different assays. Early treatment optimization based on detection of ADAs was discussed in light of the concept of transient and persistent antibodies. However, the process is still not fully understood and detection of ADAs is assay dependent and not always associated with loss of response to treatment. Formation of ADAs can be mitigated by using de-immunizing molecules, adding comedication and increasing drug exposure.

Geoff Hale (Native Antigen Company, UK) advised how the guidelines issued by the EMA and the Food and Drug Administration (FDA) are not fitted to the needs of a rapidly changing field of study. The example of polyethylene glycol (PEG) was presented with reference to the unexpectedly high frequency and titre of immunoglobulin G (IgG) anti-PEG antibodies observed in healthy populations as a consequence of the ubiquitous nature of PEG in our environment. This may have important implications in terms of the use of PEG in many drug conjugates and the utility of recombinant control agents. It was concluded that traditional cut-point approaches to the assessment of pre-existing antibodies are of little value and alternative approaches are necessary. Preventing unwanted antibodies via tolerance induction was discussed and a preview of the positive results of a proof of concept study inducing tolerance to alemtuzumab was given.

## Conclusion

The 2017 EUFEMED conference was well-attended and facilitated valuable cross-discipline interaction. It has achieved its objective of focusing on early clinical drug development in a changing regulatory environment. In closing the meeting, the president elect, Hildegard Sourgens, summarized how the topics discussed served to foster a shared appreciation of the innovative nature of the early clinical development space, and welcomed the commitment of all parties to addressing concerns over risk and improving our understanding of the challenges ahead.

## Author contributions

This work is written from an audience perspective. All authors were part of the scientific and/or organizing committee and contributed to the content of the conference. They all were able to interpret the data, critically revised the manuscript, gave final approval and agreed to be accountable for all aspects of the work in ensuring that questions related to the accuracy or integrity of any part of the work are appropriately investigated and resolved. LV, TH, and SS drafted the manuscript.

### Conflict of interest statement

The authors declare that the research was conducted in the absence of any commercial or financial relationships that could be construed as a potential conflict of interest.
